# Editorial: The psychotherapeutic framing of psychedelic drug administration

**DOI:** 10.3389/fpsyg.2023.1121234

**Published:** 2023-01-23

**Authors:** Maria Beckman, Stig Poulsen, Manoj Doss, Dea Siggaard Stenbæk

**Affiliations:** ^1^Department of Clinical Neuroscience, Center for Psychiatry Research, Stockholm Health Care Services, Stockholm County Council, Karolinska Institutet, Solna, Sweden; ^2^Department of Psychology, Faculty of Social Sciences, University of Copenhagen, Copenhagen, Denmark; ^3^Department of Psychiatry and Behavioral Sciences, Center for Psychedelic and Consciousness Research, Johns Hopkins University School of Medicine, Baltimore, MD, United States; ^4^Neurobiology Research Unit, Department of Neurology, Copenhagen University Hospital, Rigshospitalet, Copenhagen, Denmark

**Keywords:** psychedelics, psychedelic-assisted psychotherapy, psychedelic assisted therapy, set and setting, psychotherapy, therapy, integration

Research on psychedelics as treatment for psychiatric disorders have gained renewed momentum. The focus during this psychedelic resurgence has mainly been on refuting the claim that these drugs have no medical use as stated under the Controlled Substances Act (1970), by providing evidence of clinical efficacy (Horton et al., 2021), pharmacological properties (Nichols, 2016), and neurobiological effects (Madsen et al., 2019; Doss et al., 2022). Although this endeavor is well-justified, it is important to also emphasize the interdisciplinary setting required (i.e., medical and psychotherapeutic) for the clinical administration of these substances (Johnson et al., 2008). This is particularly important since both the therapeutic relationship and subjective aspects of the psychedelic experience are hypothesized as mechanisms of treatment effects (Kaelen et al., 2018; Murphy et al., 2021; Yaden and Griffiths, 2021).

Due to the profound effects of psychedelic drugs on consciousness (Mcmillan and Jordens, 2022), and since physiological risks have proven rare (Studerus et al., 2011), the main tasks of health care professionals during treatments are to alleviate psychological distress (e.g., anxiety), and facilitate beneficial effects (Johnson et al., 2008). Early psychedelic research demonstrated the importance of mindset and context, also called *set and setting*, for safe administration (Hartogsohn, 2016, 2017). With the application of set and setting protocols (i.e., psychological support protocols), or more elaborated psychotherapy models of administration, the rate of adverse responses in modern psychedelic-assisted psychotherapy (PAP) controlled trials have dropped significantly (Schlag et al., 2022). However, it is still unclear which PAP models should be considered best practice, and thus considerable heterogeneity in the psychological protocols used in clinical trials (Thal et al., 2022). Some researchers have suggested added elements of evidence-based, condition-specific psychotherapies (e.g., Sloshower et al., 2020; Horton et al., 2021). While this may lead to increased therapeutic effects, there are also arguments for more integrative approaches that take into account the unique medical and therapeutic contribution these treatments may offer.

This Research Topic brought together researchers from the psychedelic field to explore psychological models of psychedelic drug administration. The included papers span at least three levels in their approach to the topic: (1) Scoping reviews and conceptual analyses; (2) Comprehensive approaches to PAP; and (3) Specific PAP components and practices. At the first level, Cavarra et al. conducted a systematic review in which 55 papers were identified and organized according to whether the psychotherapeutic models were originally devised for psychedelics, or for traditional psychotherapeutic settings and later adopted for PAP. Common principles and differences between models and future directions are highlighted and discussed. Additionally, Bathje et al. conducted the first extensive review and concept analysis of psychedelic integration, including four models primarily based on psychotherapy, and six more spiritual/holistic models directed outside clinical settings. They also reviewed a large number of additional integration practices and activities, and incorporated the ten included models into a synthesized model of integration.

At the second level, after describing several historical and sociological influences on current psychedelic administration, Yaden et al. argued that cognitive behavioral approaches have the largest evidence-base for safety and efficacy, and therefore also the strongest rationale as the default PAP paradigm. In line with this, Mathai et al. described an acceptance and commitment therapy model for administration of esketamine, and Pots and Chakhssi presented a psilocybin-assisted compassion focused therapy for depression. However, after briefly reviewing strengths and limitations of current PAP models, Brennan and Belser argued that most of them lack adequate attention to the ethical concerns and embodied and relational elements that these treatments involve. To address this, the authors then introduced a transdiagnostic, trans-drug PAP framework.

At the third level, González et al. presented a meaning-making restorative retelling technique to process and integrate psychedelic experiences into autobiographical memory, and Sekula et al. suggested virtual reality as a possible PAP tool in their paper. Additionally, Messell et al. described a method of guided imagery and music for psychedelic interventions, and Søndergaard et al. provided evidence in favor of mindfulness-based interventions as part of future default PAP models.

Taken together, these articles make an important contribution to the present knowledge of psychotherapeutic framing of psychedelic drug administration. However, we suggest that, for a better understanding of the clinical efficacy of PAP, future trials should be designed to systematically evaluate the set and setting components of treatment, and provide detailed descriptions of all elements of the psychotherapeutic framing, including relational aspects and training of therapists.

## Author contributions

MB and DS contributed to conception and writing. All authors contributed to the editorial and approved the submitted version.
